# Effect of reducing cost sharing for outpatient care on children’s inpatient services in Japan

**DOI:** 10.1186/s13561-017-0165-3

**Published:** 2017-08-15

**Authors:** Hirotaka Kato, Rei Goto

**Affiliations:** 1 Graduate School of Economics, Kyoto University, Yoshida-honmachi, Sakyo, Kyoto, 6068501 Japan; 2 Graduate School of Business Administration, Keio University, Yokohama, Japan

**Keywords:** Heath insurance, Cost sharing, Childcare, Inpatient care, Outpatient care, I12, I18, J13

## Abstract

**Background:**

Assessing the impact of cost sharing on healthcare utilization is a critical issue in health economics and health policy. It may affect the utilization of different services, but is yet to be well understood.

**Objective:**

This paper investigates the effects of reducing cost sharing for outpatient services on hospital admissions by exploring a subsidy policy for children’s outpatient services in Japan.

**Methods:**

Data were extracted from the Japanese Diagnosis Procedure Combination database for 2012 and 2013. A total of 366,566 inpatients from 1390 municipalities were identified. The impact of expanding outpatient care subsidy on the volume of inpatient care for 1390 Japanese municipalities was investigated using the generalized linear model with fixed effects.

**Results:**

A decrease in cost sharing for outpatient care has no significant effect on overall hospital admissions, although this effect varies by region. The subsidy reduces the number of overall admissions in low-income areas, but increases it in high-income areas. In addition, the results for admissions by type show that admissions for diagnosis increase particularly in high-income areas, but emergency admissions and ambulatory-care-sensitive-condition admissions decrease in low-income areas.

**Conclusions:**

These results suggest that outpatient and inpatient services are substitutes in low-income areas but complements in high-income ones. Although the subsidy for children’s healthcare would increase medical costs, it would not improve the health status in high-income areas. Nevertheless, it could lead to some health improvements in low-income areas and, to some extent, offset costs by reducing admissions in these regions.

## Background

Many countries have adopted patient cost sharing as a way to control medical expenditures. However, the effects of patient cost sharing are yet to be completely understood. In particular, research on health impacts, the distributional consequences of cost sharing, and whether cost sharing for certain services affects the use of other services is sparse [1].

Cost sharing for a service could affect the utilization of the service itself (own-price effect) as well as reduce the utilization of complementary services or increase that of substitute services (cross-price effect). Therefore, it is important to examine both the cross-price and own-price effect. To this end, health policy studies have long debated the relationship between outpatient and inpatient services. While some insist that expanded access to primary care can reduce admissions, leading to reduced healthcare costs [2], this argument was empirically denied by the results of the RAND Health Insurance Experiment (HIE) in the mid-1970s [3, 4]. Given that hospitalization and emergency department admissions are common measures of health and health expenditures [5] and psychological biases are likely to cause patients to overweigh the immediate cost of an outpatient service relative to expected future health benefits and costs [6], it is of critical importance to empirically assess whether reduced cost sharing for outpatient care, which is often inexpensive compared to inpatient care, affects hospitalization. Nevertheless, few studies have been conducted on the cross-price effect of reducing cost sharing for outpatient care.

This study examines whether reducing cost sharing for outpatient care increases hospitalization. If outpatient and inpatient services are substitutes, expanded access to outpatient care through reduced cost sharing decreases the number of admissions. A plausible scenario is that a primary care doctor detects and successfully treats a condition that would result in hospitalization. In this case, a reduction in the number of admissions is attributed to the decrease in the number of emergency admissions and admissions due to diseases which can be managed in primary care. On the other hand, if outpatient and inpatient services are complements, expanded access to outpatient services increases the number of admissions. In this case, it is also plausible that visiting a primary care doctor results in a referral to a specialist in the hospital and a hospitalization for diagnosis or/and treatment. If this is true, the increase in the number of admissions is attributed to the expansion in the number of non-emergency admissions and admissions for diagnosis. Therefore, analysing both total admissions and admissions by type would help to more accurately reveal the relationship between inpatient and outpatient care.

Fortunately, we have an example of a suitable policy change to empirically investigate the cross-price effect. Japan’s local governments have rapidly and independently expanded subsidies for child healthcare. They have raised the upper age limit for children to access healthcare services for free or at minimal user charges. About half of them allow children to access healthcare services for free, while the other half imposes user charges—500 JPY per month as of 1 April 2012 (100 JPY equals approximately 1 USD), for example. However, the upper age limit and timing of these changes widely vary by local government. Even within a single local government, the subsidy for inpatient and outpatient care is often changed at different times. This may create exogenous variations in cost-sharing schemes for children.

Analysis of a large amount of administrative admission data shows that the subsidy for outpatient care does not significantly affect the number of overall admissions across areas. However, the effect of the subsidy on outpatient care varies by region. The outpatient care subsidy reduces the number of overall admissions in low-income areas but has the opposite effect in high-income areas, suggesting that outpatient and inpatient care are substitutes in low-income areas and complements in high-income areas.

### Literature review

The HIE is a well-known study on the effects of cost sharing on the utilization of healthcare services [3, 4]. The experiment showed a significant but modest effect in response to changes in cost sharing. In particular, children from low-income households showed decreased use of healthcare services as a result of cost sharing. However, cost sharing had no impact on the health status of the overall population. Recent empirical studies on the effect of price changes also confirmed the HIE’s findings on the own-price effect and health impact [7–10].

On the other hand, studies on the cross-price effect have obtained mixed results. Chandra et al. [11] examined a policy change which raised cost sharing for office visits and prescribed drugs for Medicare beneficiaries, and found it to be associated with a decrease in prescribed drugs and an increase in hospital admissions. Trivedi et al. [12] focused on an increase in cost sharing for outpatient care in Medicare, and showed the rise in co-payments reduced outpatient visits but increased hospitalization and inpatient days. Chandra et al. [13] analysed the impact of raising cost sharing for low-income populations and reported that inpatient and outpatient services might be substitutes, although their result was not statistically significant. By contrast, the HIE showed reduced primary care was associated with lower spending on hospitalization [4]. The Oregon Medicaid experiment also revealed that the expansion of health insurance is associated with increased hospitalization [7]. Kaestner and Sassob [14] showed that greater outpatient spending leads to higher inpatient spending. Thus, the findings on the cross-price effect are inconsistent. Moreover, since the majority of studies focus on the elderly or adults, there is little evidence on the cross-price effect for children. To our knowledge, there is little research on this issue outside the United States.

In Japan, Takaku [15] conducted a study on the impact of medical subsidies for children, specifically the effect of expanded outpatient care subsidy, using data from a nationally representative questionnaire survey conducted by the Ministry of Health, Labour and Welfare (MHLW). He found that the subsidy improved subjective health measures but did not reduce hospitalization for pre-school children, whereas it had no effect on the subjective health measures or hospitalization for school-aged children.

We believe our study complements Takaku’s [15] in two aspects. First, we use administrative admission data which cover large areas of Japan. Since Takaku [15] referenced the questionnaire survey, there is a possibility of measurement errors, such as those due to a recall bias. He focused on whether a respondent was hospitalized on the date of the survey to identify hospitalization status. Since this clearly excludes admissions before and after the survey, the use of administrative admission data can be meaningful. Second, this study examines whether the effects of the subsidy vary by regional average income. This is important because the HIE found differential effects of cost sharing.

### Institutional setting

According to official reports, all local governments subsidize out-of-pocket payments for child healthcare in Japan [16–18]. Japan’s local governments are composed of two tiers: prefectures and municipalities. Prefectures define the eligibility for the subsidy but almost all municipalities expand it. Therefore, the actual grant of the subsidy is determined by the latter. Children living in the municipality and within the age limit are granted the subsidy by their respective municipalities, although many municipalities have recently increased the eligible age [15].

The subsidized children can avail of healthcare services free of cost or must incur a minimal user charge (e.g. 500 JPY per month). Without the subsidy, preschool children bear 20% of their medical costs, and the others incur 30%. Evidently, the subsidy increases access to healthcare. Note that healthcare fees are determined by the central government, and the price of medical services is common across Japan. Since the public health insurance system in Japan covers a broad range of healthcare services and private use accounts for only about 1% of total expenditure [19], the subsidy really reduces the barriers to healthcare services.

The subsidy amount varies by government since the upper age limit considerably differs by municipality. For example, the upper age limit was 22 years in Minamifurano-cho, Hokkaido Prefecture, and 4 years in Naha-shi, Okinawa Prefecture. In addition, while some governments do not offer a subsidy to children from high-income households, others do not impose such income caps. Some governments require a minimal user charge, whereas others do not. Finally, selected governments adopt an in-kind transfer method, while others prefer a refund.

### Data

The data used in this study were taken from the Diagnostic Procedures Combination (DPC) Program. The DPC Program is a per-diem payment system for acute inpatient care and now includes a large number of hospitals for acute inpatient care. In 2012, about 1750 acute care hospitals (about 480,000 beds) participated in the DPC Program, which accounted for about 53% of all acute care beds in Japan. The DPC data include diverse information such as primary diagnosis, comorbidities, severities, treatment, admission route, admission date, discharge data, admission purpose, date of birth, and zip code of a patient. We use the data for children aged 6–18 years and hospitalized between fiscal year (FY) 2012 and 2013, since most municipalities had already subsidized healthcare for children aged up to 6 years by 2012. Note that, in Japan, the fiscal year begins on 1 April and ends on 31 March.

DPC data used here were offered by some DPC hospitals. In this study, we examined the admissions of 977 DPC hospitals which continuously provided their data for our study periods to ensure that our findings are not the result of hospital composition changes. Our data accounted for about 56% of all DPC hospitals.

A feature of DPC data is that they can provide detailed clinical information on admissions, which allows us to distinguish between emergency and non-emergency admissions, and between admissions for diagnosis and for treatments. It is important to investigate emergency admissions because these are particularly related to health.

DPC data might lead to selection bias because DPC hospitals cover only around half of acute care beds in Japan. It is true that our data include fewer patients admitted to smaller hospitals because DPC hospitals tend to be larger hospitals. However, hospitals with paediatrics tend to be still larger hospitals. According to the Survey of Medical Institutions published by the Ministry of Health, Labour and Welfare in 2013 [20], 1978 (73%) out of all 2702 hospitals with paediatrics have more than 100 beds. Therefore, the potential bias from using DPC hospital data is less serious if we focus on admissions of children.

Information on subsidy methods implemented by local governments was obtained from official reports [16–18]. These reports provide data on the upper age limits for inpatient and outpatient care subsidies: whether a local government imposed an income cap and if it applied user charges every year on 1 April. However, the reports do not offer information on the reimbursement method adopted by each municipality.

Local governments which did not have any patient aged 6–18 years during the study period were omitted. A total of 278 municipalities changed the upper age limits for outpatient care subsidy, and 1047 municipalities did not increase the limit during FY 2012–2013. We estimated 366,566 inpatients from 1390 municipalities. Table 1 presents the backgrounds of the patients examined in this study.

**Table 1:** Patient backgrounds (all admissions)

	First	Median	Average	Third	N
Age	8	12	11.715	15	366,566
Male	0	1	0.575	1	366,566
Duration of stay	3	5	8.869	9	366,566
Admission cost (JPY)	16,190	158,860	349,666.66	367,917.5	366,566
Emergency admission	0	1	0.537	1	366,566

Notes: Male = 1 if the patient is male. Emergency admission = 1 if the admission is an emergency. *JPY* Japanese yen

## Method

Municipalities have mainly expanded the upper age limits of subsidies for children. However, the age limit and timing of expansion considerably differ by municipality. We utilize these differences to identify the impact of expanding outpatient care subsidy.

We first aggregate DPC data, which are patient-level data. Using the patients’ zip codes, we identify the municipalities in which patients live. Then, we count the number of admissions for each municipality by fiscal year and create municipality-level panel data for two periods. Next, we estimate our regression model. Since the subsidy change occurs at the municipality level, this study conducts the analysis at the municipality level ×fiscal year. We treat the number of admissions in each fiscal year as the dependent variable and the upper age limits for outpatient care subsidies as of April 1 2012 and 2013 as key explanatory variables. We control for the upper age limits of inpatient care subsidies, income cap, user charge, and their regional income in each year. We also include time fixed effects and municipality-level fixed effects as explanatory variables. It is necessary that unobserved factors do not change during the specification period. However, considering that we examine the admissions of 2 years and there is no change in public healthcare fee during the period, we can assume that the important unobserved factors such as population structure and providers’ behaviour to fee-schedule are fairly stable during this short period. In addition, since we focus on DPC hospitals, patients’ choice between DPC and non-DPC hospitals should be stable during the study period. According to a public report [21], the numbers of DPC hospitals (1505 and 1496 in April 2012 and 2013, respectively), as well as those of DPC-hospital beds (479,539 and 474,981 in April 2012 and 2013, respectively), were stable during our study period. Furthermore, as mentioned, there was no change in the public fee during this period. Thus, the number of choice patients and the price, except the cost-sharing rate, were stable. Though we believe that the association between DPC and non-DPC hospitals, which affects the patients’ choices, is stable, we cannot reject the possibility of biases caused by time-variant factors affecting choice between DPC and non-DPC hospitals. Because our dependent variable is the number of admissions, we adopt a generalized linear model (GLM) and assume that the dependent variable follows a negative binomial distribution. Note that because the variance is not statistically equal to the average, we do not adopt a Poisson distribution.

It is highly likely that the effect of the subsidy varies by population group, as the HIE found heterogeneity by income and health status, and the effect of cost sharing was particularly strong among children from low-income groups. Therefore, by dividing our sample, we can examine whether the impact of reducing cost sharing varies by regional average income level of local governments. Areas whose regional average income is higher than the median regional average income are defined as high-income areas; otherwise, they are low-income areas. The regional average income of local governments is the municipal tax on per taxpayer’s income in FY 2012. Tax information was obtained from the *Survey of Local Tax* published by the Ministry of Internal Affairs and Communications.

To more clearly reveal the relationship between inpatient and outpatient care, we investigate the effects of the subsidy for each admission type. We consider two routes of admissions, two groups of primary diagnosis, and two purposes of admissions, that is, emergency and non-emergency admissions, ambulatory care sensitive condition (ACSC) and non-ACSC admission, and diagnosis and non-diagnosis admissions.

This study adopts the concept of ACSC to identify admissions sensitive to outpatient care intervention. ACSCs, which have been developed and mainly used in the United States and the United Kingdom, are conditions for which hospital admissions could have been prevented through primary care interventions [22]. We adopt the definition presented in Table 2 as the set of ACSC admissions. In our data, the number of admissions for ACSCs is 64,772, which accounts for 17.7% of all admissions.

**Table 2:** Diseases defined as ambulatory care sensitive conditions (ACSCs) and number of patients by disease

ACSCs	ICD 10 codes	N
Angina	I20, I24.0, I24.8, I24.9	64
Asthma	J45, J46	13,699
Cellulitis	L03, L04, L08.0, L08.8, L08.9, L88, L98.0	2738
Congestive heart failure	I11.0, I50, J81	171
Convulsions and epilepsy	G40, G41, R56, O15	9618
Chronic obstructive pulmonary disease	J20, J41, J42, J43, J47	3705
Dehydration and gastroenteritis	E86, K52.2, K52.8, K52.9	2746
Dental conditions	A69.0, K02, K03, K04, K05, K06, K08, K09.8, K09.9, K12, K13	273
Diabetes complications	E10.0–E10.8, E11.0–E11.8, E12.0–E12.8, E13.0–E13.8, E14.0–E14.8	918
Ear nose and throat infections	H66, H67, J02, J03, J06, J31.2	8167
Gangrene	R02	1
Hypertension	I10, I11.9	43
Influenza and pneumonia	J10, J11, J13, J14, J15.3, J15.4, J15.7, J15.9, J16.8, J18.1, J18	19,684
Iron-deficiency anaemia	D50.1, D50.8, D50.9	127
Nutritional deficiency	E40, E41, E42, E43, E55.0, E64.3	22
Other vaccine-preventable diseases	A35, A36, A37, A80, B05, B06, B16.1, B16.9, B18.0, B18.1, B26, G00.0, M01.4	687
Pelvic inflammatory disease	N70, N73, N74	335
Perforated or bleeding ulcer	K25.0–K25.2, K25.4–K25.6, K26.0–K26.2, K26.4–K26.6, K27.0–K27.2, K27.4–K27.6, K280–K282, K284–K286	318
Pyelonephritis	N10, N11, N12, N13.6	1456

Source: Purdy et al. [22]

Finally, we restrict the analysis to admissions from May to September. Since we use data on the subsidy structure as of 1 April in each year, we do not have exact data on when municipalities raised the upper age limits. In other words, we cannot deny the probability that some municipalities changed their subsidy policies during FY 2012. To address this problem and focus on the period for which we clearly understand the subsidy structure, we omit the second half of the analysed fiscal years. In addition, because April is the first month of the fiscal year, many municipalities might change their subsidies on 1 April, and admissions immediately after the policy change might be biased. Therefore, we omit admissions in April and focus on those from May to September. Table 3 reports the number of admissions for low- and high-income areas.

**Table 3:** Number of admissions in the total area, and low- and high-income areas

	Total area	Low-income areas	High-income areas
Total admissions	366,566 (100%)	56,280 (100%)	310,286 (100%)
Emergency admissions	196,943 (53.7%)	31,408 (55.8%)	165,535 (53.3%)
Non-emergency admissions	169,623 (46.3%)	24,872 (44.2%)	144,751 (46.7%)
ACSC admissions	64,772 (17.7%)	11,107 (19.7%)	53,665 (17.3%)
Non-ACSC admissions	301,794 (82.3%)	45,173 (80.3%)	256,621 (87.2%)
Admissions for diagnosis	24,760 (6.8%)	2767 (4.9%)	21,993 (7.1%)
Non-diagnosis admissions	341,806 (93.2%)	53,513 (95.1%)	288,293 (92.9%)
Admissions in May–September	169,481 (46.2%)	26,043 (46.3%)	143,438 (46.2%)

*ACSC* ambulatory care sensitive condition

## Results

Table 4 presents the descriptive statistics. The ratio of the total number of admissions in low-income areas to that in high-income areas is about 1:6, which is similar to the ratio of the populations in both areas. In low-income areas, the number of ACSC admissions is greater, and admissions for diagnosis are fewer than those in high-income areas.

**Table 4:** Descriptive statistics of estimation data

	N or median (% or IQR)		
	Total area	Low-income areas	High-income areas
Number of admission			
Total admission	37 (12–111)	22 (8–52)	80 (24–217)
Emergency admission	18 (5–57)	10 (3–27)	39 (10–118)
Non-emergency admission	17.5 (6–51)	10 (4–24)	39 (12–97)
ACSC admission	6 (1–19)	3 (1–9)	11 (3–38)
Non-ACSC admission	30 (10–90)	18 (7–43)	66.5 (19–177)
Admission for diagnosis	2 (0–7)	1 (0–3)	5 (1–14)
Non-diagnosis admission	35 (11–104)	20 (7–49)	73.5 (22–200)
Admissions from May–September	17 (5–52)	10 (4–23)	38 (10–104)
Upper age limit for inpatient care	15 (12–15)	15 (12–15)	15 (15–15)
Upper age limit for outpatient care	15 (8–15)	15 (6–15)	15 (9–15)
Income cap			
With income cap	619 (22.3%)	263 (18.9%)	356 (25.6%)
Without income cap	2161 (77.7%)	1127 (81.1%)	1034 (74.4%)
User charge			
Minimal user charge	1162 (41.8%)	578 (41.6%)	584 (42%)
Free of charge	1618 (58.2%)	812 (58.4%)	806 (58%)
Regional average income (million JPY per taxpayer)	2.67 (2.46–2.95)	2.46 (2.33–2.56)	2.95 (2.78–3.18)

Source: Data on subsidy methods (upper age limits, income cap, and user charge) were sourced from the Ministry of Health, Labour and Welfare [16–18]. Data on regional average income were sourced from *Survey of Local Tax* in 2012 and 2013 published by the Ministry of Internal Affairs and Communications. IQR stands for interquartile range. *ACSC* ambulatory care sensitive condition. *JPY* Japanese yen

Since it is important to examine the cross- and own-price effects, we present the results for both. Table 5 presents the results for overall admissions, and Table 6, for the respective effects of the subsidy by admission type.

**Table 5:** Results for the effect of raising the upper age limit on the number of admissions

	(1)	(2)	(3)
	Total area	Low-income areas	High-income areas
ln(upper age limit of outpatient care)	0.027 [0.032]	−0.200 [0.100]	0.071 [0.03]
ln(upper age limit of inpatient care)	0.028 [0.039]	0.100 [0.069]	0.015 [0.049]
N	2780	1390	1390
AIC	17,782.07	7750.513	10,012.18

Notes: Only coefficients of the main explanatory variable are reported. The dependent variable is the number of total admissions, and explanatory variables are the dummy variables for whether the local government imposed an income cap and whether it imposed a minimal user charge, regional average income, time fixed effects, and individual fixed effects. Robust standard errors are clustered at the municipality level and reported in brackets

**Table 6:** Effects of raising the upper age limit on the number of admissions by type

	(1)	(2)	(3)	(4)	(5)	(6)	(7)
	Emergency admission	Non-emergency admission	ACSC admission	Non-ACSC admission	Admission for diagnosis	Non-diagnosis admission	Restricted period
Panel A. Total area							
ln(upper age limit of outpatient care)	−0.018 [0.045]	0.079 [0.033]	−0.116 [0.08]	0.064 [0.023]	0.201 [0.048]	0.017 [0.033]	0.045 [0.043]
ln(upper age limit of inpatient care)	0.058 [0.051]	−0.008 [0.039]	0.201 [0.077]	−0.01 [0.038]	−0.071 [0.092]	0.035 [0.041]	0.006 [0.055]
N	2780	2780	2780	2780	2780	2780	2780
AIC	16,194.42	15,284.45	12,412.16	16,890.36	9032.104	17,784.96	15,290.78
Panel B. Low-income areas							
ln(upper age limit of outpatient care)	−0.249 [0.12]	−0.118 [0.096]	−0.506 [0.224]	−0.06 [0.07]	0.195 [0.273]	−0.212 [0.101]	−0.166 [0.081]
ln(upper age limit of inpatient care)	0.061 [0.089]	0.125 [0.07]	0.295 [0.158]	0.028 [0.059]	0.327 [0.239]	0.086 [0.073]	0.19 [0.108]
AIC	6624.11	6444.212	4964.5	7385.877	3453.593	7677.529	6597.879
Panel C. High-income areas							
ln(upper age limit of outpatient care)	0.031 [0.046]	0.098 [0.04]	−0.01 [0.078]	0.081 [0.024]	0.191 [0.048]	0.064 [0.03]	0.079 [0.044]
ln(upper age limit of inpatient care)	0.065 [0.064]	−0.034 [0.048]	0.186 [0.093]	−0.02 [0.047]	−0.166 [0.101]	0.031 [0.051]	−0.051 [0.06]
N	1390	1390	1390	1390	1390	1390	1390
AIC	9073.718	8462.417	7259.917	9481.202	5593.513	9968.935	8942.465

Notes: Only coefficients of the main explanatory variable are reported. The dependent variable is the number of admissions by type, and the explanatory variables are the dummy variables for whether the local government imposed an income cap and whether it imposed minimal user charges, regional average income, time fixed effects, and individual fixed effects. Robust standard errors are clustered at the municipality level and reported in brackets. *ACSC* ambulatory care sensitive condition

### Total number of admissions

Column (1) in Table 5 shows the effect of raising the upper age limit for the subsidy on the overall number of admissions in the total area (low- and high-income areas), and columns (2) and (3) do so for low- and high-income areas, respectively. The row showing details on ln(upper age limit of outpatient care) presents the change in total admissions as a result of expanding the upper age limit for outpatient care subsidy (cross-price effect). We find that the estimate is not significant for the total area. However, the estimate varies by regional income level: it is significantly negative in low-income areas (−0.2) but significantly positive in high-income areas (0.07). That is, raising the upper age limit for the outpatient care subsidy from 12 to 15 years (25% increase) could decrease the number of admissions by 5% in low-income areas but increase it by 2% in high-income areas.

The row showing details on ln(upper age limit of inpatient care) indicates the change in the total number of admissions caused by an increase in the upper age limit for the inpatient care subsidy (own-price effect). The estimates of the own-price effect are not significant in all columns.

### Admission by type

Next, we show whether the effect of the subsidy varies by admission type. Panel A in Table 6 shows the results for the sample of the total area, while Panel B and Panel C do the same for low-income areas and high-income areas, respectively. Columns (1) and (2) show the effects on emergency and non-emergency admissions. The estimates of ln(upper age limit of outpatient care) for non-emergency admissions are significantly positive in the total area and high-income areas, whereas in the low-income areas, the estimate for emergency admissions is significantly negative. The own-price effects are not significant in both columns.

Columns (3) and (4) report the effect on ACSC and non-ACSC admissions. With regard to the total area and high-income areas, raising the upper age limit for outpatient care significantly increases non-ACSC admissions. By contrast, in low-income areas, the subsidy for outpatient services significantly reduces ACSC admissions. The estimates for ln(upper age limit of inpatient care) of ACSC admissions are significantly positive in the total area and high-income areas.

Columns (5) and (6) present the effects on diagnosis and non-diagnosis admissions. The estimates of ln(upper age limit of outpatient care) for admissions for diagnosis are significantly positive in the total area and high-income areas, whereas those for non-diagnosis admissions are significantly negative in the low-income areas. All the estimates for ln(upper age limit of inpatient care) are not significant.

Column (7) reports the result of the analysis in which we count only the admissions from May to September. The estimates in Table 5 and column (8) of Table 6 are not markedly different. The point estimates of ln(upper age limit of outpatient care) are similar in both low- and high-income areas. In addition, the estimate in low-income areas is also significant; however, that for high-income areas is not significant at the 5% level but is significant at the 10% level (Table 6).

## Discussion

We find that the outpatient care subsidy does not have a significant impact in the total area. However, the effects vary by regional income level: while the outpatient care subsidy decreases the number of admissions in low-income areas, it increases those in high-income areas. In addition, as expected, when the subsidy reduces the number of admissions, the number of emergency and ACSC admissions decreases, and when it increases the number of admissions, those for non-emergency and admissions for diagnosis increase.

It may be not surprising that the own-price effect is not significant because our admission data constitute patients with acute conditions, whose demand for inpatient services is probably less sensitive to price change.

Our results lead us to conclude that outpatient and inpatient services are substitutes in low-income areas but complements in high-income ones. Thus, even within the same country and time period, the relationship between inpatient and outpatient services is sensitive to regional income level. This possibly explains why previous research on the relationship between inpatient and outpatient care presents mixed results.

In addition, we assess the impact of the subsidy. In high-income areas, despite the increase in admissions for diagnosis, the number of emergency admissions does not decrease. Since the number of emergency admissions has been used as a reliable and common measure of health [5], it appears that the recent dramatic expansions of the subsidy increase medical expenditures but may not improve the health levels of children in high-income areas. However, in low-income areas, the reduction of cost sharing for outpatient care decreases the number of admissions, including emergency admissions. Although the magnitude of the effect of the subsidy on hospitalization is not as large, this implies that reducing cost sharing for outpatient care provides some health improvements, and its cost could be somewhat offset by the declining number of admissions in low-income areas. In addition, it suggests that people in low-income areas may underuse outpatient care in the absence of the subsidy.

There are numerous reasons people with varying income levels are affected differently by cost sharing [13]. First, low-income groups may simply be more responsive because they face a greater budget constraint. Second, such groups may underestimate the marginal benefits of healthcare services and thereby, underuse them probably because of their lower education levels. Third, they are more likely to suffer from chronic illness. These reasons explain why regional income level strongly affects the relationship between inpatient and outpatient care.

The contributions of this study are as follows. First, this study adopted rich administrative admission data, making it possible to avoid biases from a questionnaire survey and to allow a more accurate estimation of admissions. Since the public health insurance system in Japan covers a broad range of healthcare services, the administrative data contain almost all information on inpatient care usage. In addition, because the data cover most areas in Japan, we could report results which are not specific to a municipality or health insurer. Second, this study focuses on children. Few studies conducted on the cross-price effect have done so from the viewpoint of child admissions. Since hospitalization is highly expensive and a reliable measure of health [5], and childhood health is known to have a long-term impact [23], the findings of this study are of critical importance. The results of the cross-price effects have implications for a more suitable cost-sharing design.

The policy implication of this study is as follows. The subsidy for outpatient care increases the usage of medical services but may not achieve apparent health improvement. However, the subsidy in low-income areas may improve children’s health by reducing the number of emergency admissions and ACSC admissions. Hence, the current rapid expansion of subsidy for outpatient care in Japan cannot be supported. Eligibility of subsidy for outpatient care should be more carefully examined; provision of subsidy depending on the regional income level could be more efficient. Implementing this policy may be difficult if municipalities emulate other municipalities [24, 25], which could lead to excessive yardstick competition on generous cost-sharing and deteriorations of financial conditions of particular municipalities in financial difficulties. However, the central government in Japan has the power to control each municipality to a certain extent by monitoring the use of strategic subsidy. Thus, the central government may be able to reduce the problem arising from competition on cost-sharing rate among municipalities. Furthermore, it has already begun to pay more attention to cost-containment policy for healthcare. The central government could play an important role to more efficiently design the subsidy policy in the future.

However, our study is subject to certain limitations. First, our data consist of DPC hospitals treating acute patients. Therefore, we are not sure whether our results are applicable to non-acute patients although our data include non-emergency admissions and admissions for diagnosis as shown in Table 3. In addition, we cannot access the total impact on the utilization of outpatient care. Second, we cannot directly solve the potential endogeneity of policy change. Municipalities whose admissions tend to vary might raise the upper age limit of the subsidy. Because we do not have proper instrumental variables, we cannot completely address this problem. However, many municipalities have stated that the key objective of the subsidy is to prevent a decline in birth rate, and access to health care and low birth rate are administered by different sections in many municipalities. Therefore, this endogeneity is probably not a serious problem. Nevertheless, if municipalities decide their subsidies based on the decisions of other municipalities, the change in cost sharing is not independent, which may create a bias in our results. We do not overcome this potential problem.

Third, our results might be biased due to the confounding variables which cannot be controlled by fixed effects: we cannot completely control the bias arising from the change in the population or population structure which is associated with subsidy policy. For example, the subsidy policy could induce patient relocations: it is likely that parents with sick children move to an area whose government offers a larger subsidy. Patient relocation inevitably alters the population number and its structure, thereby affecting the number of admissions. Although the incentive to relocate may not be strong since the eligibility for the subsidy is temporary (as children will eventually outgrow the target age of subsidies (15)) and the relocation problem could not explain the decrease in the number of admissions in low-income areas, our results might be biased to some extent because of the above-mentioned problem.

Finally, as mentioned, the subsidy structure is not completely understood in this study. Owing to the lack of data, we are unaware of the exact time each government expanded its subsidy and of the reimbursement method (in-kind transfer or refund). Although this may cause biases, since the results of the restricted analysis do not markedly differ from the main findings, and the change in reimbursement methods does not completely explain why some admission types are affected and others are not, we believe that the biases do not fully erode the key conclusions of this study. However, it is desirable to more accurately understand the finer details of the subsidy policy. These issues will be addressed in future research.

## Conclusions

We find that reduced cost sharing for outpatient care decreases the number of admissions in low-income areas, while it increases those in high-income areas. These results suggest that outpatient and inpatient services are substitutes in low-income areas but complements in high-income ones. Our study makes a significant contribution to the literature because research on cross-price effects is limited. Our findings expand our understanding of the relationship between inpatient and outpatient care, and show the importance of considering variation in the effects of cost sharing by subpopulation. This could help implement more effective health insurance design and policy.
